# Regional adiposity and markers of inflammation in pre-school age children

**DOI:** 10.1038/s41598-018-33054-1

**Published:** 2018-10-12

**Authors:** Kerri Z. Delaney, Catherine A. Vanstone, Hope A. Weiler, Sylvia Santosa

**Affiliations:** 1 Department of Health, Kinesiology, and Applied Physiology, Concordia University, Montreal, Quebec Canada; 2 Metabolism, Obesity, Nutrition Lab, PERFORM Centre, Concordia University, Montreal, Quebec Canada; 3 Centre de recherche - Axe maladies chroniques, Centre intégré universitaire de santé et de services sociaux du Nord-de-l’Ile-de-Montréal, Hôpital du Sacré-Coeur de Montréal, Montréal, PQ Canada; 4 School of Human Nutrition, McGill University, Ste-Anne-de-Bellevue, Quebec Canada

## Abstract

In adults, upper body fat partially increases metabolic disease risk through increasing systemic inflammation. Our objective was to determine if this relationship exists in preschool-aged children. A subset of children (n = 71, 35 males), 3.7 ± 1.0 y, were studied from n = 515 children recruited from randomly selected daycares in Montréal, QC. According to WHO charts for 2–5 y, 49 children were healthy weight (HW) and 21 were overweight (OW). Adiposity was determined through dual-energy x-ray absorptiometry. Blood concentrations of C-reactive protein (CRP) and tumour necrosis factor alpha (TNFα) were determined via enzyme-linked immunosorbent and multiplex assays, respectively. OW children had higher (p = 0.03) android:gynoid ratio 0.50 ± 0.09 compared to HW children 0.56 ± 0.12, indicating excess fat was predominantly stored in the abdominal depot. CRP was higher (p = 0.01) in OW children 1.45 ± 2.02 mg/L compared to HW 0.74 ± 1.38 mg/L. Percent fat was correlated with CRP (r = 0.32; p < 0.01) and TNFα (r = 0.25; p = 0.04) concentrations. CRP also correlated with android adiposity (r = 0.24; p = 0.04) and TNFα correlated with gynoid adiposity (r = 0.24; p = 0.04). We observed that greater adiposity is associated with higher systemic inflammation in pre-school aged children. Future longitudinal studies are needed to understand the long term consequences of excess total and regional body fat in young children.

## Introduction

Evidence shows that the increase in the incidence of overweight and obesity in adults is also occurring in preschool age children^1^. The most recent population-level assessment reporting on body mass index (BMI) in Canadian 2–5 year olds found approximately 17% were overweight or obese^1^. At this time, research examining the implications of carrying excess adipose tissue has been dominated by studies in adult populations with results from these studies often being extrapolated to children. However, these extrapolations come with limitations, as there are several physiological differences between children and adults. Studies conducted in children are required to accurately assess the implications of obesity in this population.

In adults, several studies have found a relationship between increased adipose tissue mass and systemic inflammatory markers^2^. This relationship is especially salient to upper body/android adipose tissue which has been described to be associated with greater inflammation than lower body/gynoid adipose tissue^3,4^. The greater inflammation observed in individuals with android obesity has been shown, in part, to contribute towards the greater risk for developing metabolic diseases including cardiovascular disease and type 2 diabetes^5,6^. A number of causes have been proposed for why this phenomenon occurs, most of which culminate in an increase in pro-inflammatory cytokines such as tumor necrosis factor-alpha (TNFα) and C-reactive protein (CRP)^7–9^.

Although the positive relationship between increases in adipose tissue and systemic inflammation has been well established in adults^10–13^ and teenagers^14–17^, few studies have examined this relationship in youth 6–12 years^18,19^ and young children 2–5 years^20,21^. Furthermore, to our knowledge, there has yet to be a study examining the association between regional adipose tissue mass and inflammation in children 2–5 years of age. The objective of this study was to determine if a relationship between whole body and regional adiposity and markers of inflammation exists in pre-school age children. We hypothesize that systemic inflammation will increase with greater whole body mass and that there will be a stronger association between inflammation and android adiposity compared to gynoid adiposity.

## Methods

### Study Population and Protocol

This study examined a subset of preschoolers (n = 71) who underwent whole body fan-beam clinical dual-energy X-ray absorptiometry (DXA) scans. This subset was obtained from a larger sample of pre-school age children (n = 514, 2–5 years old) who were recruited from a random sample of licensed daycares in the greater Montreal area (n = 77). The daycares included represented 10% of the daycares registered with the Ministère de la Famille et des Ainès, (n = 733), which represents 91% of the Greater Montreal regions. Pamphlets outlining the details of the study were sent home to 8440 parents. Of these parents, 625 agreed to be contacted. After screening for eligibility over the telephone, 570 children were initially recruited to participate in the study. Children were included if they were healthy and term born. Children were excluded if they had a disease causing disturbances of bone metabolism, known or suspected chronic illness of childhood, use of medication known to affect bone metabolism in the past 3 months, history of prior treatments for vitamin D deficiency, and severe anemia. Due to absences, lack of cooperation on the assessment days or insufficient sample volume, a final sample size of 514 children was obtained. A detailed description of participant inclusion has been previously described^22^.

Anthropometric measures and capillary blood samples were collected by a registered nurse who attended each daycare for the purposes of this study. All 514 children were invited for a DXA scan conducted by a trained personal at the Mary Emily Clinical Nutrition Research Unit, McGill University. Of the 514 children, 71 agreed to the DXA scan. When anthropometric and descriptive characteristics where compared, the 71 children who underwent a DXA scan were not significantly different from the other 443 children except for in age, with the children who underwent a DXA scan being older (p = 0.001) than those who did not. All 71 DXA scans were successfully completed with no movement breaks in the images. Reports of recent physician visits were examined to exclude children with a recent illness and resulting elevated inflammation. Of the 71 children, two were excluded for having abnormally high levels of CRP and TNFα defined by greater than five standard deviations from the mean.

### Ethics

The McGill University Faculty of Medicine Institutional Review Board approved this study and the secondary use of collected data. A parent or legal guardian provided written informed consent before the study. All measures were conducted in accordance to guidelines and regulations.

### Anthropometric Measures and Body Composition

All anthropometric and body composition measures were taken while children were wearing light clothing (shorts and T-shirt) and no shoes. Height and weight was measured using standard procedures by a registered nurse at each daycare and by trained personnel at the Mary Emily Clinical Nutrition Research Unit on the day of each DXA scan^22^. Height was measured with a portable stadiometer (Seca 213, Seca Medical Scales and Measuring Systems, Hamburg, Germany) and body weight was measured with a digital scale (Home Collection 63-8711-0, Trileaf Distribution, Toronto, ON, Canada).

BMI was calculated as weight (kg)/ height (m)^2^. Children were divided by BMI percentiles according to World Health Organization charts for Canadian boys and girls, aged 2–19 years^23^. According to these charts, children were categorized as underweight (<3 percentile, n = 1), healthy weight (3–85 percentile, n = 49), or overweight (>85 percentile, n = 21) with no children falling into the obese category (>99 percentile). If children were classified using the CDC sex-specific BMI-for-age growth charts n = 6 children (3 males) would have been classified as obese.

To determine whole body and regional measures of adiposity, children underwent a whole body DXA scan (APEX version 13.2:3; Hologic, 2005, 4500A Discovery Series, Bedford, MA). After careful placement of whole body regions, the software automatically generates android and gynoid regions. The android region is defined with a lower limit of the iliac crest and upper limit set at 20% of the distance from the iliac crest to the base of the mandible. The gynoid region is defined with an upper limit of the iliac crest to an inferior distance that is 1.5 times the height of the android region.

### Biochemical Assessment

A registered nurse collected 1 ml capillary blood samples by finger lance. All blood samples were collected between 0800 h and 1200 h and stored on ice between time of collection and arrival to the laboratory. At the laboratory, samples were centrifuged at 3000 *g* and 4 °C for 20 minutes. Plasma aliquots were stored at −80 °C until time of analysis.

Plasma CRP concentrations were measured using enzyme-linked immunosorbent assays (R&D Systems Quantikine, Minneapolis, Minn., USA; DRG International Inc., Mountainside, N.J., USA; Bachem, Bubendorf, Switzerland). Plasma TNFα concentration was measured using multiplex assay for small-volume samples (catalog No. HMHMAG- 34 K, Millipore, Billerica, Mass., USA). There was no lower limit of detection (LOD) in the analysis of CRP. The LOD for TNFα with this assay was 0.3 pg/mL. If values were below the LOD they were reported as LOD/2 rather than zero. The average confidence interval on triplicate measurements (n = 30) was 6.7% for CRP and 9.8% for TNFα.

### Statistics

Data were first checked for normality using Shapiro-Wilks tests. Where possible non-parametric data was normalized using log transformations (total fat mass and android fat mass). CRP was not normally distributed and transformed data failed to meet the conditions of normality; thus non-parametric tests were used for this variable. Independent sample t-tests (parametric) and Mann-Whitney U (non-parametric) were used to compare differences between healthy and overweight children. A univariate analysis of variance (ANOVA) with Fisher’s least significant difference (LSD) post hoc was then used to test for differences in sex by weight categories. To test for the effect of covariates; sex and age, an analysis of covariance (ANCOVA) was run.

To determine if correlations existed between regional and whole body measures of adiposity and markers of inflammation Pearson (parametric) and Spearman (non-parametric) correlations were conducted. To test for covariates; sex and age, a partial correlation test was used. Further covariates, race and household income, were examined however, are not reported as they did not affect the statistical analyses. Data were further split into healthy and overweight categories and correlations were run again to determine if the strength of any existing correlations changed. Studies that examine CRP and TNFα in children are limited. A study by Alvarez *et al*.^19^ measured CRP and TNFα in overweight vs. lean children 7–12 y. Based on their measurements a mean difference and a standard deviation (SD) of 0.93 mg/L and 0.45 ng/mL, respectively in CRP, and 0.59 ng/mL and 0.66 ng/mL concentrations in TNFα is expected. Therefore, we would need 20–21 participants per group to detect a significant difference at α = 0.05 and β = 0.80. All data were analyzed with SPSS v22 (Cary, NC). All data are presented as mean ± SD with p < 0.05 defined as significantly different.

## Results

### Participant Characteristics

Of the 71 children included in the study, the average age was 3.7 ± 1.0 years and 35 were male. The average BMI-for-age z-score was 0.04 ± 0.95 with the majority (n = 49) of children being healthy weight, between the 3rd and 85th percentiles. The average android:gynoid ratio and percent fat was 0.52 ± 0.11 and 27.2 ± 5.1%, respectively.

When children were split into overweight and healthy weight categories, males comprised 57% (n = 12) of overweight and 47% (n = 23) of healthy weight children (Table 1). Healthy weight children were older than those who were overweight (p = 0.02; Table 1). BMI z-score, total fat mass and percent fat were significantly higher in overweight children (p < 0.01; Table 1). All measures of regional adiposity (android fat mass and gynoid fat mass) were significantly higher in overweight children (p < 0.01; Table 1). Overweight children had a significantly greater android:gynoid ratio of 0.56 ± 0.12 compared to healthy weight children at 0.50 ± 0.09 (p = 0.03). Differences in the android:gynoid ratio between healthy and overweight children were no longer significant when sex, and age were controlled for.

**Table 1:** Participant characteristics by weight category.

Variables	Healthy Weight 3rd-85th BMI° (n = 49)	Overweight > 85th BMI° (n = 21)	p-value
Sex (M, F)	47%, 53%	57%, 43%	
Age (years)	3.9 ± 0.9	3.3 ± 1.1	0.02
BMI z-score	−0.37 ± 0.64	1.10 ± 0.54	<0.01
Percent Fat (%)	25.5 ± 3.8	31.3 ± 5.7	<0.01
Total Fat Mass (kg)^†^	4.3 ± 0.7	5.7 ± 1.2	<0.01
Android Fat Mass (g)^†^	189 ± 50	285 ± 116	<0.01
Gynoid Fat Mass (g)	777 ± 160	1042 ± 226	<0.01
Android:Gynoid	0.50 ± 0.09	0.56 ± 0.12	0.03
CRP (mg/L)^a^	0.74 ± 1.38*	1.45 ± 2.02	0.01
TNFα (ng/L)	6.85 ± 2.88*	7.94 ± 3.18	0.06

Data are mean ± SD. P-value shown for Independent samples t-test or ^a^Mann-Whitney U unadjusted for sex and age. ^†^log transformed, *n = 47.

### Markers of Inflammation

Circulating concentration of CRP was significantly greater in overweight compared to healthy weight children at 1.45 ± 2.02 mg/L and 0.74 ± 1.38 mg/L, respectively (p = 0.01; Table 1). When results were adjusted for sex they remained significant, however, when age was added to the model they were no longer significant. On average concentration of TNFα was greater in overweight children, with a trend (p = 0.06) towards significance, at 7.94 ± 3.18 ng/L compared to 6.85 ± 2.88 ng/L in healthy weight children (Table 1). This trend no longer existed when sex, and age were controlled for. Overweight males had significantly higher levels of TNFα than overweight females, and healthy weight males and females (p < 0.05).

### Relationships between Whole and Regional Adiposity and Inflammation

**Figure 1:**
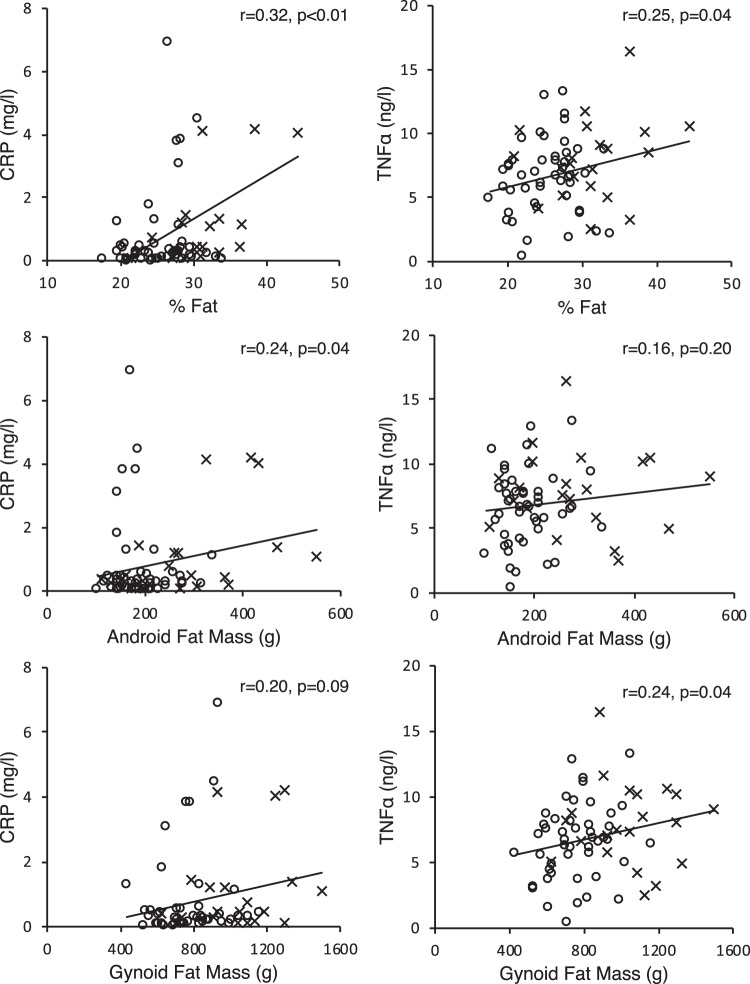
Pearson (TNFα) and Spearman (CRP) correlations between inflammatory markers and percent fat, android fat mass and gynoid fat mass. n = 68, -represents a correlation in the whole group; o represents healthy weight; x represents overweight.

Both CRP (r = 0.32; p < 0.01) and TNFα (r = 0.25; p = 0.04) were positively correlated with percent fat (Fig. 1). There were no correlations between other whole body measures of adiposity (BMI, total fat mass) and markers of inflammation (Table 2). Android fat mass positively correlated with CRP (r = 0.24; p = 0.04) but not TNFα. In contrast, gynoid fat mass positively correlated with TNFα (r = 0.24; p = 0.04) but not CRP. All results remained significant when age and sex were controlled for. When children were divided into healthy and overweight categories, the strength of the correlation between CRP and percent fat increased to moderate at r = 0.62 (p < 0.01) in overweight children. A negative correlation (r = −0.47, p < 0.05) between fat free mass and CRP was also observed in overweight children.

**Table 2:** Relationship between inflammatory markers and measures of adiposity.

Variables	CRP (mg/L)^a^	TNFα (ng/L)
BMI (kg/m^2^)	0.09	0.18
Total Fat Mass (kg)^†^	0.17	0.18
% Fat (%)	0.32**	0.25*
Android Fat Mass (g)^†^	0.24*	0.16
Gynoid Fat Mass (g)	0.20	0.24*
Android:Gynoid	0.09	−0.004

Data are Pearson (TNFα) or Spearman (CRP) correlation coefficients: *p < 0.05; **p < 0.01; n = 68. ^†^log transformed. Controlled for age and sex.

## Discussion

To our knowledge this is the first study to examine the association between systemic markers of inflammation and regional and whole body adiposity in children 2–5 years of age. Our findings show children who were overweight had significantly higher levels of CRP than healthy weight counterparts. Furthermore, both CRP and TNFα positively correlated with percent fat mass with the correlation in CRP being stronger in overweight children. There were no clear associations between regional measures of adiposity and systemic markers of inflammation, with both the android and gynoid depot correlating with one marker of inflammation but not both. Before sex and age were controlled for, overweight children had a significantly greater android:gynoid ratio indicating excess adipose tissue was predominantly stored in the android region.

Higher levels of inflammation have previously been found in overweight youth^14–19^ and adults^10–13^ when compared to healthy weight individuals however, few studies have examined this relationship in young children under the age of 5 years^20,21^. In adults, the increase in systemic inflammation resulting from excess adipose tissue has been linked to the development of type 2 diabetes and cardiovascular disease^24,25^. Circulating concentration of CRP is now used in clinical settings to determine the risk of a cardiovascular event in adults; a concentration of <1.0 mg/L indicating low risk, 1–3 mg/L moderate risk, and >3.0 mg/L high-risk^26^. In youth and children (1–17 years), there is yet to be a clinically establish concentration for elevated CRP. Similarly, there is no clinically established risk level of elevated TNFα in children or adults.

In our study, we found that compared to healthy weight counterparts, children who were overweight had increased concentrations of TNFα and double the concentration of circulating CRP. Our results are consistent with the study by Skinner *et al*.^20^ whose examination of NHANES data showed that very obese 3–5 year old children had a greater prevalence of high CRP. In our study, the positive age and sex adjusted correlations between percent fat mass and CRP and TNFα further supports that adipose tissue contributes to increases in these inflammatory markers. At this time, it cannot be said if elevated levels of systemic inflammation at a young age causes significant or lasting damage to the vascular system that would contribute to the development of cardiovascular disease in later life. Presently there is a lack of both cross sectional and longitudinal data following children throughout their development to know the effect of elevated adipose tissue mass and systemic inflammation in early years.

Utilizing DXA scans allowed for the accurate measure of whole body and regional body composition in study participants. Previous studies in children in the 2–5 year age range have relied on inferior methods to measure adiposity such as skin fold assessments, bio-electrical impedance analysis or waist: hip radio^27^. Overweight children had a higher android:gynoid ratio than healthy weight children. The greater android:gynoid ratio indicates that children were predominantly storing excess adipose tissue in their abdominal depot. If this trend continues throughout their development, they could be at an increased risk for the early onset of metabolic disease^28,29^.

We found no distinct associations between android or gynoid adiposity and makers of inflammation as positive associations were observed between CRP and android fat mass, and TNFα and gynoid fat mass. In adults, the predominant storage of excess weight in the abdominal depot compared to the gynoid depot has been correlated with increased levels of circulatory inflammatory markers and increased risk of metabolic disease^5,6^. One reason for our observations may be that body morphology varies greatly between and within children as they grow and develop through these early years^30^. Likely, the relationship between elevated inflammation and abdominal adiposity does not occur until after the age of 5 as found by Staiano *et al*.^31^.

Since this study is cross sectional in design we are unable to delineate whether the differences in regional adipose tissue caused changes in inflammatory markers. The relationships we found in this study are important first steps towards understanding how adiposity affects inflammation in children. In our study, as defined by the World Health Organization charts for boys and girls, there were not any children with obesity. It is plausible that including children with obesity would have resulted in clearer associations between regional adiposity and inflammation. It should be noted, however, that there were significant differences in regional adipose tissue depot mass between the children with healthy and overweight, and there was a large range in regional fat depot masses within our study participants. According to the latest Statistics Canada report, the prevalence of overweight/obesity in Quebec children in the 2–5 age range is 26.1%^32^.

In this study, we were unable to delineate between upper body subcutaneous and visceral adipose tissue mass. Though the direct quantification of visceral adipose tissue via MRI would have been ideal, the cost, access, and most importantly, difficulty in conducting an MRI scan in this age group was prohibitive. The use of the DXA in our study provided accurate measurement of total and regional fat depots. This is the first study of this scale to examine whole body DXA scans and inflammatory risk factors in young children. Future longitudinal studies should examine the long term risk associated with the weight driven increase in systemic inflammatory markers in young children. Specifically, the age of onset and the years of exposure to elevated levels of systemic inflammation should be examined. By furthering our knowledge in this area of study, we will be able to better understand the long term consequences of childhood obesity.

In conclusion, our study found young children in the 2–5 y age range had a weight associated increase in systemic inflammatory markers. Future longitudinal studies are needed to understand the long term consequences of excess total and regional body fat in young children. Our results show, even in this age range, that children are negatively affected by carrying excess adipose tissue perpetuating the importance of early prevention programs.

## Data Availability

The datasets generated during and/or analyzed during the current study are not publicly available due to the inclusion of individuals who are minors but are available from the corresponding author on reasonable request.
